# Estimating the healthcare costs of overweight and obesity amongst Australian children

**DOI:** 10.1111/imj.70432

**Published:** 2026-05-05

**Authors:** Winnie Chen, Anagha Killedar, Mohammad Nure Alam, Kirsten Howard, Louise A Baur, Alison Hayes

**Affiliations:** ^1^ Leeder Centre for Health Policy, Economics and Data, Faculty of Medicine and Health University of Sydney Sydney New South Wales Australia; ^2^ Menzies School of Health Research Charles Darwin University Darwin Northern Territory Australia; ^3^ Sydney School of Public Health, Faculty of Medicine and Health University of Sydney Sydney New South Wales Australia; ^4^ NHMRC Centre of Research Excellence in the Early Prevention of Obesity in Childhood (EPOCH) University of Sydney Sydney New South Wales Australia; ^5^ Specialty of Child and Adolescent Health, Sydney Medical School University of Sydney Sydney New South Wales Australia

**Keywords:** childhood obesity, paediatric obesity, healthcare costs, health economics, Australia

## Abstract

This study aimed to describe healthcare utilisation and costs associated with excess weight amongst Australian children (mean age 11 years). We analysed self‐reported data from the Child Health CheckPoint study. From a healthcare provider perspective, children with overweight and obesity incurred annual excess costs of AUD +$140 and +$379 (+$117 and $328 adjusted) respectively compared to those in the healthy weight category. These findings suggest obesity is a significant driver of healthcare costs in children and support the economic argument for reducing childhood obesity.

Childhood obesity is a complex condition with multifaceted causes. In Australia, approximately 28% of children aged 5 to 17 years are affected by overweight or obesity.^1^ Worldwide, the prevalence of childhood overweight and obesity has increased steadily since the 1990s and is projected to increase further by 2030.^2^, ^3^ Childhood obesity is a major contributor to poorer physical health across the life course. Obesity is associated with increased risk of chronic conditions such as cardiovascular disease and type 2 diabetes and premature mortality.^4^, ^5^ Furthermore, childhood obesity can negatively impact an individual's psychological health and wellbeing.^6^

Childhood obesity is costly, both in terms of increased direct healthcare costs in childhood, as well as increased lifelong costs associated with obesity‐related comorbidities.^7^, ^8^ At a population level, these economic impacts are substantial. Carrello *et al*. estimated that achieving the Australian National Obesity Strategy's goal of 5% reduction in childhood overweight and obesity could result in lifetime savings of over $7 billion (2030 AUD).^9^

In Australia, several studies to date have estimated the cost differences between children with healthy weight versus overweight and/or obesity.^7^ However, most have been limited to out‐of‐hospital expenditures, derived from Medicare Benefits Scheme (MBS) and Pharmaceutical Benefits Scheme (PBS) datasets.^10^, ^11^, ^12^ One costing study analysed linked hospital‐based costs, but only for children aged 2 to 5 years within the trial.^13^ Consequently, for children of all other ages, a knowledge gap remains regarding cost differences by weight status, particularly with respect to hospital‐based costs. Accurate and comprehensive costs are important for informing health policy and assessing cost‐effectiveness of childhood obesity strategies. Therefore, the aim of this study was to characterise healthcare utilisation and associated healthcare costs amongst Australian children, and to compare these costs by weight status (healthy weight, overweight and obese).

This was a cross‐sectional study, including all children within the Child Health CheckPoint module, of the ‘Growing Up in Australia: The Longitudinal Study of Australian Children’ (LSAC) study.^14^ Ethical approval was granted from the University of Sydney Human Research Ethics Committee (2022/HE000699). Direct healthcare costs were described, both from a healthcare funder and patient perspective. We report costs in $AUD 2024–2025 unless otherwise specified. Where applicable, costs were adjusted to $AUD 2024–2025 using Australian Institute of Health and Welfare deflators.^15^ A 12‐month time horizon is used, consistent with responses from the cross‐sectional study.

LSAC is a nationally representative longitudinal study of Australian children, which began in 2004 with over 10 000 children. LSAC data collection waves have been conducted every 2 years; now in its tenth wave, there are approximately 20 years of follow‐up data for included participants.^16^ The Child Health CheckPoint (referred to as CheckPoint hereafter) was a once‐off module focussing on physical health, nested between waves 6 and 7 of the LSAC study. Conducted between 2015 and 2016, CheckPoint collected data for children in the birth cohort (‘B cohort’) of the LSAC study, who were approximately 11 years old at the time of data collection.^14^

Children were classified into three weight categories (healthy weight, overweight, obesity) according to anthropometric measurements taken in LSAC wave 6 by a trained interviewer. Height and weight were used to calculate body mass index (BMI), which was transformed to the relevant BMI *Z* scores for age and sex. As per World Health Organization cut offs, underweight and healthy weight were classified as body mass index (BMI) *Z* score ≤ 1; overweight as BMI *Z* score ≥ 1 and < 2; obesity as BMI *Z* score ≥ 2.^17^ From the LSAC wave 6 data we also used additional demographic variables including sex, socioeconomic position (SEP) and cultural and linguistic language background. SEP is a variable within LSAC that combines measures of parental income, education and occupation status as a *Z* score.^18^ These LSAC variables have been described in detail previously.^19^, ^20^

The CheckPoint study provided additional data not previously available within LSAC on self‐reported healthcare utilisation and out‐of‐pocket costs. This included questions on hospital‐based and out‐of‐hospital healthcare utilisation over the past 12 months. Healthcare provider costs were estimated based on publicly available unit costs for each service type (Table 1). For each type of healthcare service accessed, CheckPoint had a corresponding question on out‐of‐pocket costs. We focussed on services with a clear public funding component and excluded those where most respondents (>80%) paid out‐of‐pocket. We further categorised out‐of‐hospital services into general practitioner (GP), dental, medical specialist and allied health categories.

**Table 1:** Unit costs of healthcare services, cost per visit (AUD)

Type	Healthcare provider cost	OOP cost as per CheckPoint study, mean (SD)	Reference for healthcare provider cost
Hospital‐based services†			
Inpatient admission	7973	N/A	Independent Health and Aged Care Pricing Authority^23^
ED presentation	1252	N/A	Independent Health and Aged Care Pricing Authority^23^
Hospital outpatients	515	N/A	Independent Health and Aged Care Pricing Authority^23^
Community‐based services‡			
GP	44	34 (39)	MBS^24^ – item 23
Dentist	72	120 (570)	MBS^24^– item 75800
Medical specialist—paediatrician	234	125 (99)	MBS^24^– item 135
Medical specialist—psychiatrist	234	105 (92)	MBS^24^– item 289
Medical specialist—ENT	76	237 (377)	MBS^24^– item 109
Allied health—psychologist	99	90 (224)	MBS^24^– item 80000
Allied health—physiotherapy	62	43 (107)	MBS^24^ – item 10960
Allied health—optometry	66	150 (112)	MBS^24^ – item 10905
Allied health—dietitian	62	100 (84)	MBS^24^ – item 10954
Allied health—speech pathology	62	34 (56)	MBS^24^ – item 10974

Abbreviations: ED, emergency department; ENT, ear, nose and throat; GP, general practitioner; MBS, Medicare Benefits Scheme; OOP, out‐of‐pocket.

† OOP costs are assumed to be zero for hospital‐based services.

‡ MBS rebate at 100% for GP, 75% for dental and medical specialists and 85% for allied health services – reflecting government contributions.

Statistical analysis was conducted in StataBE 18^21^ and R 4.2.2.^22^ The primary analysis described associations between weight category and total mean annual healthcare provider costs. As a secondary analysis, we also compared out‐of‐pocket costs across weight categories. Differences between weight categories were assessed using the Kruskal–Wallis test for non‐parametric data, with statistical significance set at *P* < 0.05. To estimate adjusted differences in healthcare provider costs by weight category, we used two‐part models. The two‐part model used logistic regression for binary outcome of whether any costs were incurred, followed by a generalised linear model with log link and Gaussian family and log link to predict annual healthcare costs for each weight status, adjusting for relevant demographic covariates (sex, SEP *Z* score, language background). We assessed for potential interactions of weight status with SEP and language background in the adjusted model.

Table S1 presents the characteristics of the CheckPoint study participants by weight status. We included 1874 parent–child pairs within the CheckPoint study, with a mean child age of 11 years. Of these, 67% were in the healthy weight category, 21% in the overweight category and 12% in the obesity category. There was a socioeconomic gradient across the weight categories, with the lowest socioeconomic status (SEP *Z* scores) for children in the obesity weight category.

Healthcare utilisation: Table 2 shows the count of healthcare utilisation episodes over 12 months across various healthcare services. Statistically significant differences were observed in hospitalisations and GP visits across weight categories, with utilisation of these services increasing from healthy to obesity weight categories.

**Table 2:** Healthcare utilisation by weight category, mean (SD) annual visits per child

Type	Overall, *N* = 1874	Healthy weight, *n* = 1255	Overweight, *n* = 389	Obesity, *n* = 230	*P* value†
Inpatient admission	0.0454 (0.2745)	0.0390 (0.2695)	0.0514 (0.2915)	0.0696 (0.2716)	0.02
ED presentation	0.26 (0.70)	0.25 (0.68)	0.26 (0.69)	0.34 (0.78)	0.10
Hospital outpatients	0.17 (0.72)	0.16 (0.71)	0.22 (0.80)	0.15 (0.67)	0.48
GP	2.11 (4.82)	2.05 (5.59)	2.22 (2.66)	2.27 (2.58)	0.01
Dentist	1.61 (2.73)	1.65 (3.18)	1.57 (1.60)	1.44 (1.20)	0.83
Medical specialists	0.23 (0.87)	0.22 (0.86)	0.20 (0.69)	0.32 (1.15)	0.45
Allied health	1.62 (4.82)	1.39 (3.38)	1.80 (4.63)	2.56 (9.48)	0.40

Abbreviations: ED, emergency department; GP, general practitioner; SD, standard deviation.

† Kruskal‐Wallis rank sum test.

For healthcare provider and out‐of‐pocket costs, overall, children across the study incurred a mean cost of $1493 (SD 2716) over 12 months, with 70% incurred in healthcare provider costs, and 30% in out‐of‐pocket costs (Fig. S1). In terms of healthcare provider costs, over half of the costs per child were attributable to hospital‐based services (Table 3, Fig. 1). For out‐of‐pocket costs, the highest costs per child related to medical specialist costs.

**Table 3:** Healthcare provider cost by weight status, mean (SD) annual cost per child (AUD)

Type	Overall, *N* = 1874	Healthy weight, *n* = 1255	Overweight, *n* = 389	Obesity, *n* = 230	*P* value†
Inpatient admission	283 (1713)	244 (1681)	321 (1819)	434 (1694)	0.02
ED presentation	327 (876)	310 (859)	322 (869)	425 (972)	0.10
Hospital outpatients	89 (373)	84 (364)	111 (414)	78 (344)	0.48
GP	85 (193)	82 (224)	89 (106)	91 (103)	0.01
Dentist	119 (202)	122 (235)	116 (118)	106 (89)	0.83
Medical specialists	41 (168)	43 (179)	32 (129)	48 (162)	0.48
Allied health	98 (296)	81 (178)	115 (292)	162 (627)	0.32
Total cost	1042 (2469)	966 (2464)	1106 (2509)	1345 (2405)	0.01

Abbreviations: ED, emergency department; GP, general practitioner; MBS, Medicare Benefits Scheme; OOP, out‐of‐pocket; SD, standard deviation.

† Kruskal‐Wallis rank sum test.

**Figure 1:**
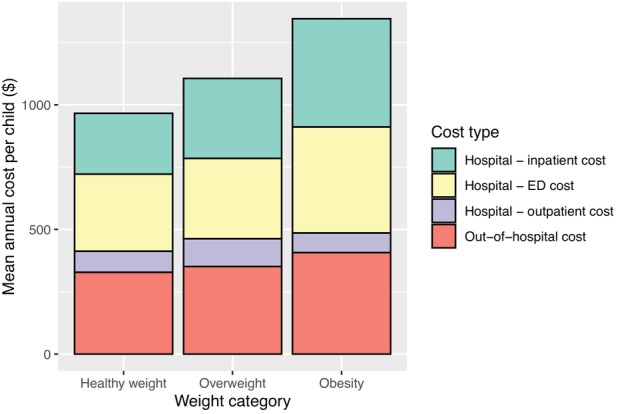
Healthcare provider costs, by weight category, mean annual cost per child (AUD). This figure shows the proportion of healthcare provider costs, according to cost type (green—hospital inpatient, yellow—hospital ED, purple—hospital outpatient, red—out‐of‐hospital costs), by weight category. ED, emergency department.

For healthcare costs by weight category, Figure 1 shows that healthcare provider costs increased from those in the healthy weight category ($966 (SD $2464)) to the overweight category ($1106 (SD 2509)) to the obesity category ($1345 (SD $2405)) per child over 12 months (*P* < 0.01). This was primarily driven by higher inpatient hospitalisation costs in the overweight and obesity categories compared with the healthy weight category (Table 3). Out‐of‐pocket costs were not statistically different across weight categories (Tables S2 and S3).

After adjusting for sex, SEP and language background, predicted excess annual healthcare provider costs were +$117 (+$140 unadjusted) for the overweight category and +$328 (+$379 unadjusted) for the obesity category, compared with the healthy weight category (Table S4). The two‐part model is presented in full in Table S5. No significant interactions were found between weight category and SEP scores, and weight category and language background, indicating that the relationship between weight status and healthcare costs did not differ according to these demographic factors.

## Discussion

To date, most Australian studies examining the economic impacts of childhood overweight and/or obesity have focussed on out‐of‐hospital costs. Given the substantial cost of hospital‐based services, our findings are important in characterising the broader healthcare cost burden of overweight and obesity. We observed that children in the overweight and obesity categories had an annual healthcare cost increase of $140 and $379 respectively compared with healthy weight children. These differences persisted, even when adjusted for sex, socioeconomic position and language background. We also considered out‐of‐pocket costs and found no statistically significant differences across weight categories, which may reflect Australia's healthcare system where many paediatric services are publicly subsidised. Our work represents a novel contribution to the literature, as most international studies have not incorporated out‐of‐pocket costs in their analyses.^7^

Our observed cost differences across weight categories are larger than previously reported in Australian LSAC studies, likely because earlier work focussed on examining MBS and/or PBS costs only. Despite a younger cohort in the Australian Healthy Beginnings Trial (under 5 years) compared with those in this study (age approximately 11 years), Hayes *et al*. also found that hospital‐based costs contributed to the majority of excess costs.^13^ This is consistent with international studies examining excess costs associated with childhood obesity.^23^

This study has several limitations. Firstly, although the CheckPoint study provides valuable information on healthcare utilisation and out‐of‐pocket costs, it is cross‐sectional in nature and reliant on self‐reported questionnaire data. Unit costs were estimated from best available sources but were not directly available. Future linkage of administrative datasets, particularly inpatient admission diagnosis‐related groups, would provide improved estimates of hospital utilisation and costs over a longer period. Secondly, whilst the initial LSAC cohort is nationally representative, the CheckPoint sub‐study may be subject to selection bias. CheckPoint participants were also of a narrow age range. Taken together, these factors may limit the generalisability of our cost findings to all Australian children. Importantly, there may be differences in healthcare provision by jurisdiction, or by rurality, which would be important to explore in detail in future studies. Thirdly, the observed association between childhood obesity and increased healthcare costs may partly reflect the impact of medical conditions associated with obesity, such as endocrine disorders and genetic syndromes,^24^ as well as other clinical or social factors. Individual‐level clinical data would be required to better characterise clinical drivers and presentations associated with increased healthcare costs. Lastly, it has been a decade since the CheckPoint study was conducted, with new research needed to reflect potential changes in childhood obesity epidemiology and patterns of healthcare use. For example, with the introduction of the National Disability Insurance Scheme (NDIS) in 2013, funding models and associated costs to access allied health services have changed for NDIS‐eligible children.

In conclusion, our study found that childhood obesity was associated with notable increases in healthcare provider costs. Even without accounting for long‐term health and economic impacts of obesity across the life course, these short‐term cost differences support the economic argument for obesity prevention and management.

## Supporting information


**Figure S1** Healthcare provider and out of pocket costs by weight category, mean annual cost per child (AUD).
**Table S1.** Child characteristics by weight category
**Table S2.** Out‐of‐pocket cost by weight category, mean (SD) annual cost per child (AUD)
**Table S3.** Total healthcare cost by weight category, mean (SD) annual cost per child (AUD)
**Table S4.** Unadjusted and adjusted healthcare provider costs by weight category, mean annual cost per child (AUD)
**Table S5.** Two part model for estimating annual healthcare provider costs

## Data Availability

The data that support the findings of this study are available from Australian Data Archive. Restrictions apply to the availability of these data, which were used under license for this study. Data are available from https://ada.edu.au/ with the permission of Australian Data Archive.
