# Supplementary material for: Estimating the healthcare costs of overweight and obesity amongst Australian children
Source: Intern Med J. 2026 May 5;56(6):1081–6. doi: 10.1111/imj.70432 (PMC13288316; doi:10.1111/imj.70432)
Supplement: Supplementary file 1 — Figure S1 Healthcare provider and out of pocket costs by weight category, mean annual cost per child (AUD). Table S1. Child characteristics by weight category Table S2. Out‐of‐pocket cost by weight category, mean (SD) annual cost per child (AUD) Table S3. Total healthcare cost by weight category, mean (SD) annual cost per child (AUD) Table S4. Unadjusted and adjusted healthcare provider costs by weight category, mean annual cost per child (AUD) Table S5. Two part model for estimating annual healthcare provider costs [file IMJ-56-1081-s001.pdf]

Supplementary material

Title: Estimating the healthcare costs of overweight and obesity among Australian children

Authors: Winnie Chen, Anagha Killedar, Mohammad Nure Alam, Kirsten Howard, Louise A Baur, Alison Hayes

Contents:

- Figure S1: Healthcare provider and out of pocket costs by weight category, mean annual cost per child (AUD)
- Table S1: Child characteristics by weight category
- Table S2: Out-of-pocket cost by weight category, mean (SD) annual cost per child (AUD)
- Table S3: Total healthcare cost by weight category, mean (SD) annual cost per child (AUD)
- Table S4: Unadjusted and adjusted healthcare provider costs by weight category, mean annual cost per child (AUD)
- Table S5: Two part model for estimating annual healthcare provider costs

Figure S1: Healthcare provider and out of pocket costs by weight category, mean annual cost per child (AUD)

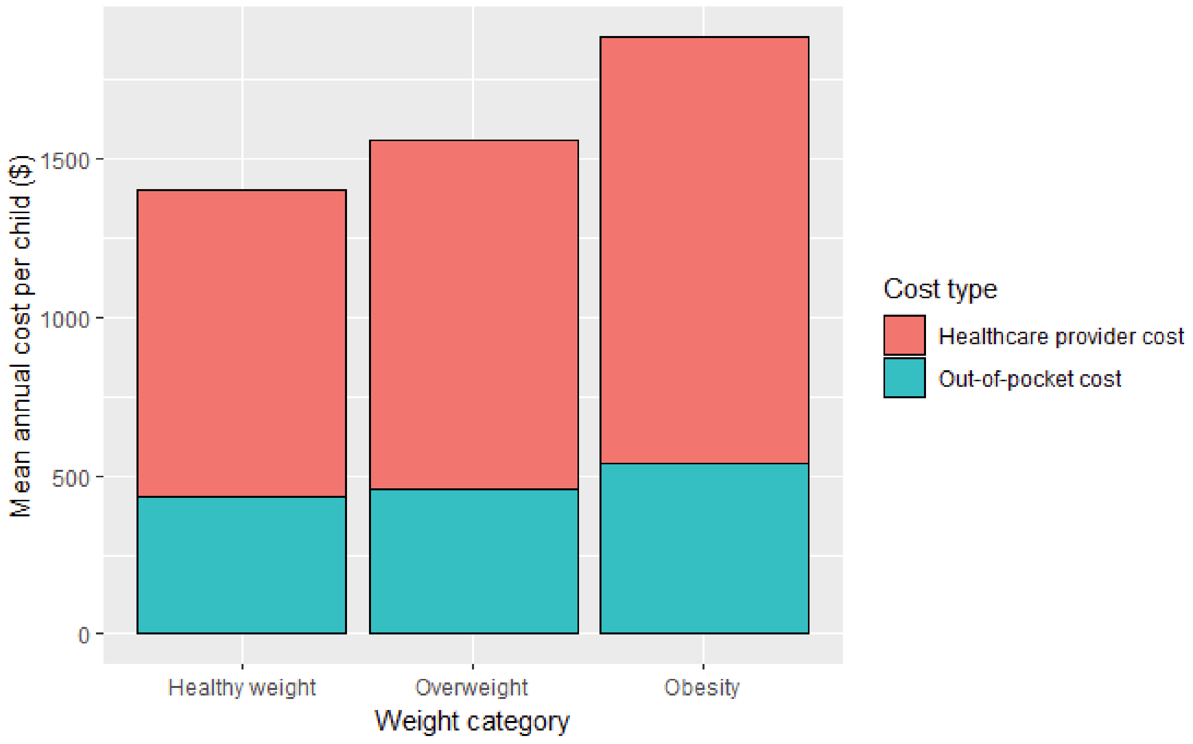

Figure S1 shows proportion of costs paid by healthcare provider (red) versus out-of-pocket (blue), by weight category.

Table S1: Child characteristics by weight category

**Characteristic**	**Overall N = 1,874**	**Healthy weight N = 1,255**	**Overweight N = 389**	**Obesity N = 230**	**p-value†**
Age, years (SD)	10.91 (0.33)	10.91 (0.33)	10.89 (0.34)	10.98 (0.33)	<0.01
Sex, n (%)					0.03
• Male	955 (51%)	624 (50%)	195 (50%)	136 (59%)	
• Female	919 (49%)	631 (50%)	194 (50%)	94 (41%)	
Socioeconomic position (SEP) Z-score (SD)	0.17 (0.99)	0.25 (0.99)	0.06 (1.01)	-0.06 (0.88)	<0.01
Socioeconomic position (SEP) quintile, n (%)					<0.01
• 1 (Lowest)	375 (20%)	215 (17%)	95 (24%)	65 (28%)	
• 2	375 (20%)	247 (20%)	87 (22%)	41 (18%)	
• 3	374 (20%)	249 (20%)	69 (18%)	56 (24%)	
• 4	375 (20%)	264 (21%)	67 (17%)	44 (19%)	
• 5 (Highest)	375 (20%)	280 (22%)	71 (18%)	24 (10%)	
Cultural and linguistic background, n (%)					0.34
• English speaking background	1,512 (81%)	1,015 (81%)	305 (78%)	192 (83%)	
• Non English speaking background	328 (18%)	219 (17%)	77 (20%)	32 (14%)	
• Aboriginal and/or Torres Strait Islander	34 (1.8%)	21 (1.7%)	7 (1.8%)	6 (2.6%)	

†Kruskal-Wallis rank sum test, except for cultural and linguistic background which used Fisher's exact test.
Abbreviations: SEP – socioeconomic position (score).

Table S2: Out-of-pocket cost by weight category, mean (SD) annual cost per child (AUD)

Type	Overall N = 1,874	Healthy weight N = 1,255	Overweight N = 389	Obesity N = 230	p-value†
GP	73 (166)	71 (193)	77 (92)	79 (89)	0.01
Dentist	191 (325)	196 (378)	186 (190)	171 (143)	0.83
Medical specialists	34 (135)	31 (122)	32 (115)	52 (209)	0.42
Allied health	154 (446)	136 (345)	160 (382)	239 (846)	0.35
Total cost	452 (626)	434 (598)	455 (491)	541 (914)	0.12

†Kruskal-Wallis rank sum test.
Abbreviations: GP – general practitioner.

Table S3: Total healthcare cost by weight category, mean (SD) annual cost per child (AUD)

**Type**	**Overall N = 1,874**	**Healthy weight N = 1,255**	**Overweight N = 389**	**Obesity N = 230**	**p-value†**
Healthcare provider cost	1,042 (2,469)	966 (2,464)	1,106 (2,509)	1,345 (2,405)	0.01
Out-of-pocket cost	452 (626)	434 (598)	455 (491)	541 (914)	0.12
Grand total cost‡	1,493 (2,716)	1,400 (2,685)	1,561 (2,723)	1,887 (2,842)	0.01

†Kruskal-Wallis rank sum test.
‡Grand total costs included healthcare provider and out-of-pocket costs.

Table S4: Unadjusted and adjusted healthcare provider costs by weight category, mean annual cost per child (AUD)

	**Healthy weight**	**Overweight**	**Obesity**
Unadjusted cost (mean, 95% CI)	966 (830 to 1,102)	1,106 (957 to 1,355)	1,345 (1,034 to 1,656)
Difference vs healthy weight	-	+140	+379
Adjusted cost (mean, 95% CI)†	972 (836 to 1,108)	1089 (996 to 1604)	1300 (996, 1604)
Difference vs healthy weight	-	+117	+328

†Adjusted for sex, socioeconomic position (SEP score), and language background.

Abbreviations: CI – confidence interval.

Table S5: Two part model for estimating annual healthcare provider costs

	Logistic regression			GLM		
Variable	OR	95% CI (OR)	p-value	Cost ratio	95% CI (cost ratio)	p-value
Sex (vs male)						
Female	1.32	0.87 to 2.01	0.19	0.91	0.74 to 1.12	0.37
Weight category (vs healthy weight)						
Overweight	0.92	0.56 to 1.51	0.74	1.12	0.87 to 1.46	0.37
Obesity	1.42	0.69 to 2.92	0.34	1.32	1.01 to 1.72	**<0.05**
Socioeconomic position (SEP Z-score)	1.25	1.03 to 1.52	**0.03**	0.86	0.78 to 0.95	**<0.01**
Language background (vs Aboriginal and/or Torres Strait Islander)						
English-speaking background	1.25	0.29 to 5.38	0.77	0.87	0.48 to 1.57	0.65
Non English-speaking background	0.50	0.11 to 2.23	0.37	0.63	0.32 to 1.24	0.18

Two-part model for estimating annual healthcare provider cost. First part using logistic regression, second part using GLM with log link and Gaussian family. Full model presented above including sex, weight category, socioeconomic position (SEP Z-score), and language background. Bold indicates p<0.05.

Abbreviations: CI – confidence interval; OR – odds ratio; SEP – socioeconomic position (score)
